# Deep learning-based detection of seedling development

**DOI:** 10.1186/s13007-020-00647-9

**Published:** 2020-07-30

**Authors:** Salma Samiei, Pejman Rasti, Joseph Ly Vu, Julia Buitink, David Rousseau

**Affiliations:** 1 Laboratoire Angevin de Recherche en Ingénierie des Système (LARIS),UMR INRAe IRHS, Université d’Angers, Angers, France; 2 Institut de Recherche en Horticulture et Semences-UMR1345, Université d’Angers, INRAe, Institut Agro, SFR 4207 QuaSaV, Beaucouzé, France; 3 Department of Big Data and Data Science, École d’ingénieur Informatique et Environnement (ESAIP), Angers, France

**Keywords:** Seedling development, Deep learning, Kinetic

## Abstract

**Background:**

Monitoring the timing of seedling emergence and early development via high-throughput phenotyping with computer vision is a challenging topic of high interest in plant science. While most studies focus on the measurements of leaf area index or detection of specific events such as emergence, little attention has been put on the identification of kinetics of events of early seedling development on a seed to seed basis.

**Result:**

Imaging systems screened the whole seedling growth process from the top view. Precise annotation of emergence out of the soil, cotyledon opening, and appearance of first leaf was conducted. This annotated data set served to train deep neural networks. Various strategies to incorporate in neural networks, the prior knowledge of the order of the developmental stages were investigated. Best results were obtained with a deep neural network followed with a long short term memory cell, which achieves more than 90% accuracy of correct detection.

**Conclusion:**

This work provides a full pipeline of image processing and machine learning to classify three stages of plant growth plus soil on the different accessions of two species of red clover and alfalfa but which could easily be extended to other crops and other stages of development.

## Background

A specificity of plants is their continuous capability to metamorphose during their lifetime. This process is characterized by the kinetics of ontological development stages, i.e., stages that occur in a definite order. In this article, we focus on some of these connected steps of a plant’s life at the seedling level. The period from seed germination in the soil to the development of the first true leaf is crucial for the plant. During this time, the seedling must determine the appropriate mode of action based on its environment to best achieve photosynthetic success and enable the plant to complete its life cycle. Once the seedling emerges out the soil, it initiates photomorphogenesis, a complex sequence of light-induced developmental and growth events leading to a fully functional leaf. This sequence includes severe reduction of hypocotyl growth, the opening of cotyledons, initiation of photosynthesis, and activation of the meristem at the shoot apex, a reservoir of undifferentiated cells that will lead to the formation of the first leaf [1]. The molecular mechanisms regulating these time-based events involves profound reprogramming of the genome that is challenging to study in field situation because the heterogeneity of the seedling population must be taken into account. It is essential to understand this seedling development process from an agronomic point of view because the seedling establishment is critical to crop yield. Uneven emergence timing, for instance, is associated with lower yields and poor farmer acceptance.

In this context, time-lapse imaging is a valuable tool, accessible at a rather low-cost [2–5], for documenting plant development and can reveal differences that would not be apparent from a sole endpoint analysis. At the seedling level where plants have simple architectures, such time-lapse imaging can be done from top view to provide an efficient solution for seedling vigor assessments and monitoring of seedling growth. While some statistical tools transferred from developmental biology exists to perform time-to-event analysis [6], a current bottleneck [7] lay in the automation of the image analysis. A recent revolution occurred in the field of automated image analysis with deep neural networks [8], which have shown their universal capability to address almost any image processing challenges with high accuracy. This revolution also benefits plant imaging [9], and it is currently a timely topic to adapt these tools, which came from the artificial intelligence community to specific topics of interest in plant sciences. In this article, we propose an entire pipeline based on deep learning dedicated to the monitoring of seedling growth.

Seedling growth monitoring with computer vision has received considerable attention in the literature including [10–24]. It is therefore important to locate our proposition with these related works. While each article of this literature deals with the quantification of some aspects of the early stages of plant development, it includes a large variety of approaches behind the word seedling. Several studies consider germination and seedling growth measurements in vitro, using plastic boxes or paper towel [10–17, 21], which enable the monitoring of radicle emergence (germination) or organ growth (seedling growth). Others, like in this article, used soil-based sowing systems, where seedling emergence and early developmental events of the aerial part can be determined under more realistic agronomical conditions [19, 22–26]. Reported approaches to monitor seedling from the top view in the soil are effective for a large set of crops, mainly at the emergence level, i.e., seedling counting to determine stand establishment [19, 23–26], or estimating early plant vigor by spectral imaging or measuring the leaf area index of the small plants [19, 22, 26]. As most related work, deep learning has been applied to the problem of seedling detection and segmentation [24]. By contrast with our work, this has been performed at a fixed stage of development. Here we propose to push forward the detection of the early seedling developmental stages to be able to monitor the kinetics of early seedling development in the soil from cotyledon emergence until the development of the first real leaf. We propose to tackle this task of seedling kinetics monitoring, for the first time to the best of our knowledge, with a deep learning-based approach.

Spatio-temporal approaches in deep-learning have been extensively developed in computer vision for video processing [27] but has so far been very rarely applied in plant imaging [28] (for growth prediction). As most related work in spatio-temporal processing [2] proposed a graph-based method for detection and tracking of tobacco leaves at the late stage of the plant growth from infrared image sequences. This study was not based on deep learning and was applied on later stage of development than seedling. In the last similar approach [20], a feature-based machine learning algorithm distinct from deep learning was developed to detect two stages of heading and flowering of wheat growth.

In this article, we investigate, for the first time to the best of our knowledge in plant imaging, how the existing methods of spatio-temporal deep learning, can incorporate time-dependency in sequences of images to solve the problem of monitoring the developmental kinetics. While the proposed method is of general value for developmental biology, its performance is assessed on the specific use case of seedlings of red clover and alfalfa imaged from top view.

## Materials and method

The proposed plant method includes four main items: (i) The imaging system developed to create (ii) the dataset, which needs to benefit from (iii) pre-processing before investigating (iv) various approaches for the detection of developmental stages of seedling growth based on deep learning methods.

### Imaging system

**Fig. 1:**
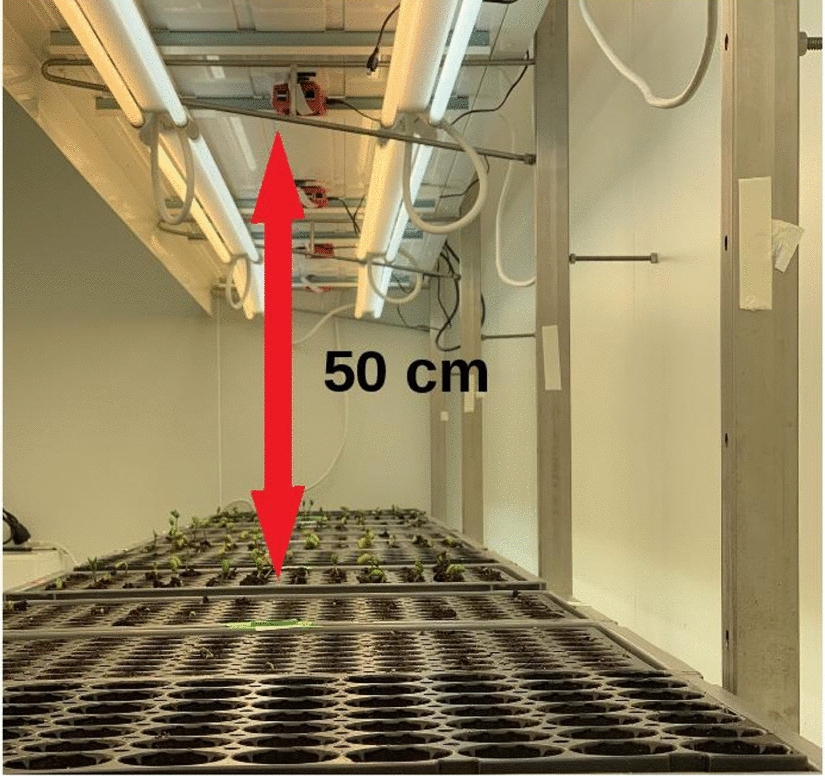
Imaging system installed in a growth chamber

A set of minicomputers (as described in [3]) connected to RGB cameras with a spatial resolution of 3280 by 2464 pixels was used to image seedlings from the top view as illustrated in Fig. 1. The distance of 50 cm was chosen to allow the observation of 2 trays of 200 pots per camera.

### Dataset

Seedling establishment was recorded for 3 experiments using seed lots from different accessions of red clover (Trifolium pratense) (experiment 1) and alfalfa (Medicago sativa) (experiments 2 and 3). Each experiment consisted of 70 trays with 200 pots in which 50 seeds of four accessions were sown. Soil pots were hydrated to saturation for 24h after which excess water was removed. After 24h, seeds were sown at a depth of 2 cm, and trays were placed in a growth chamber at $20^\circ \hbox {C}/16^\circ \hbox {C}$, with 16 h for photoperiod at $200 \upmu M m^{-2} s^{-2}$. The soil was kept humid throughout the experiment.

**Fig. 2:**
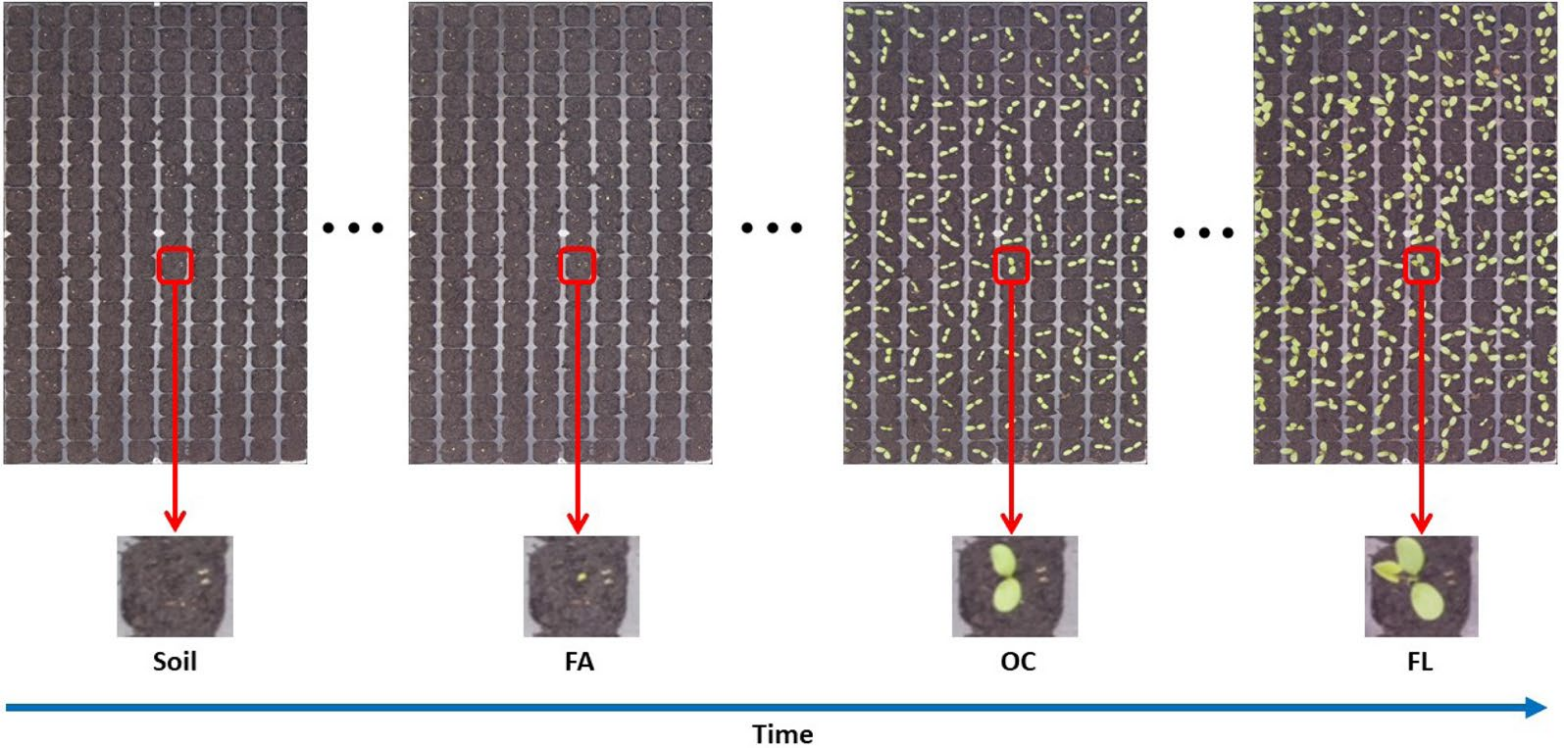
An overview of the time-lapse collected for this work. Upper row, view of a full tray with 200 pots from the top view. Lower row, a zoom on a single pot at each stage of development to be detected from left to right: soil, the first appearance of the cotyledon (FA), opening the cotyledons (OC) and appearance of the first leaf (FL)

Each experiment took two weeks with a time-lapse of 15 minutes. In total, the database consists of 42000 temporal sequences of RGB images of size $89 \times 89 \times 3$ pixels where each temporal sequence consists of 768 individual images. During day time, images were captured while images during night times were automatically discarded due to the absence of illumination. An example of images from the database is shown in Fig. 2. Among all temporal sequences, images of 3 randomly selected trays were manually annotated by a plant expert from the first experiment (red clover species) and 2 trays from the second experiment (alfalfa species). This ground-truth annotation consisted of four classes: soil, the first appearance of the cotyledon (FA), the opening of the cotyledon (OC), and the appearance of the first leaf (FL). The algorithms proposed in this article for timing detection of seedling emergence following these four stages of development were trained, validated and tested against this human-annotated ground-truth. In order to avoid cross sampling, we considered images of the trays of the red clover for training (two trays) and validation (one tray) datasets. The testing dataset consisted of images of the remaining two trays from the alfalfa. Table 1 provides a synthetic view of the data set used for training and testing of the models.

**Table 1:** Description of the split of the annotated data set for training models

	Species	No. of trays	No. of pots in each tray	No. of temporal sequences	Total No. of images
Training dataset	Red clover	2	200	400	307,200
Validation dataset	Red clover	1	200	200	153.600
Testing dataset	alfaalfa	2	200	400	307,200

**Fig. 3:**
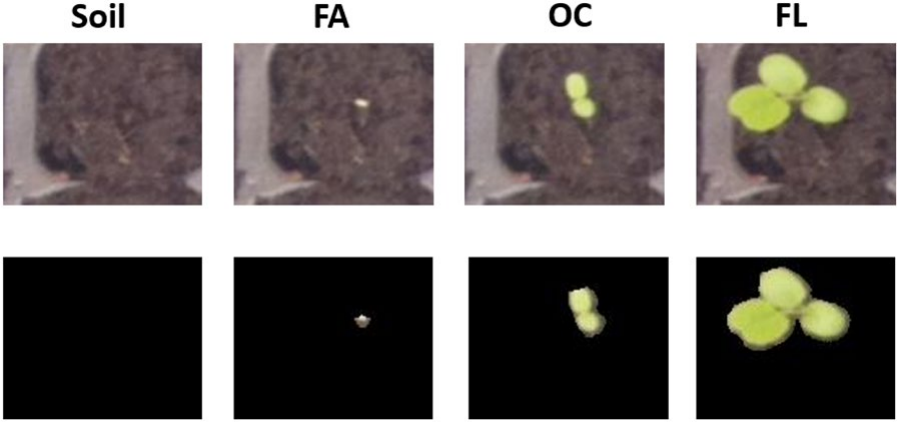
Two different types of data used in training and testing. Up: Original images, Down: Images without background

Raw images were then sent to pre-processing before being applied to the deep learning method investigated in this study. A filtered variant of the raw images was also created where the soil background was removed from images. This filter was produced by applying a color filter on images in the HSV color domain to keep the green range of images in the Hue channel. This strategy was found robust because the soil used during the experiment was the same, and that lighting was kept constant. Figure 3 shows an example of images with and without background.

### Pre-processing

**Fig. 4:**
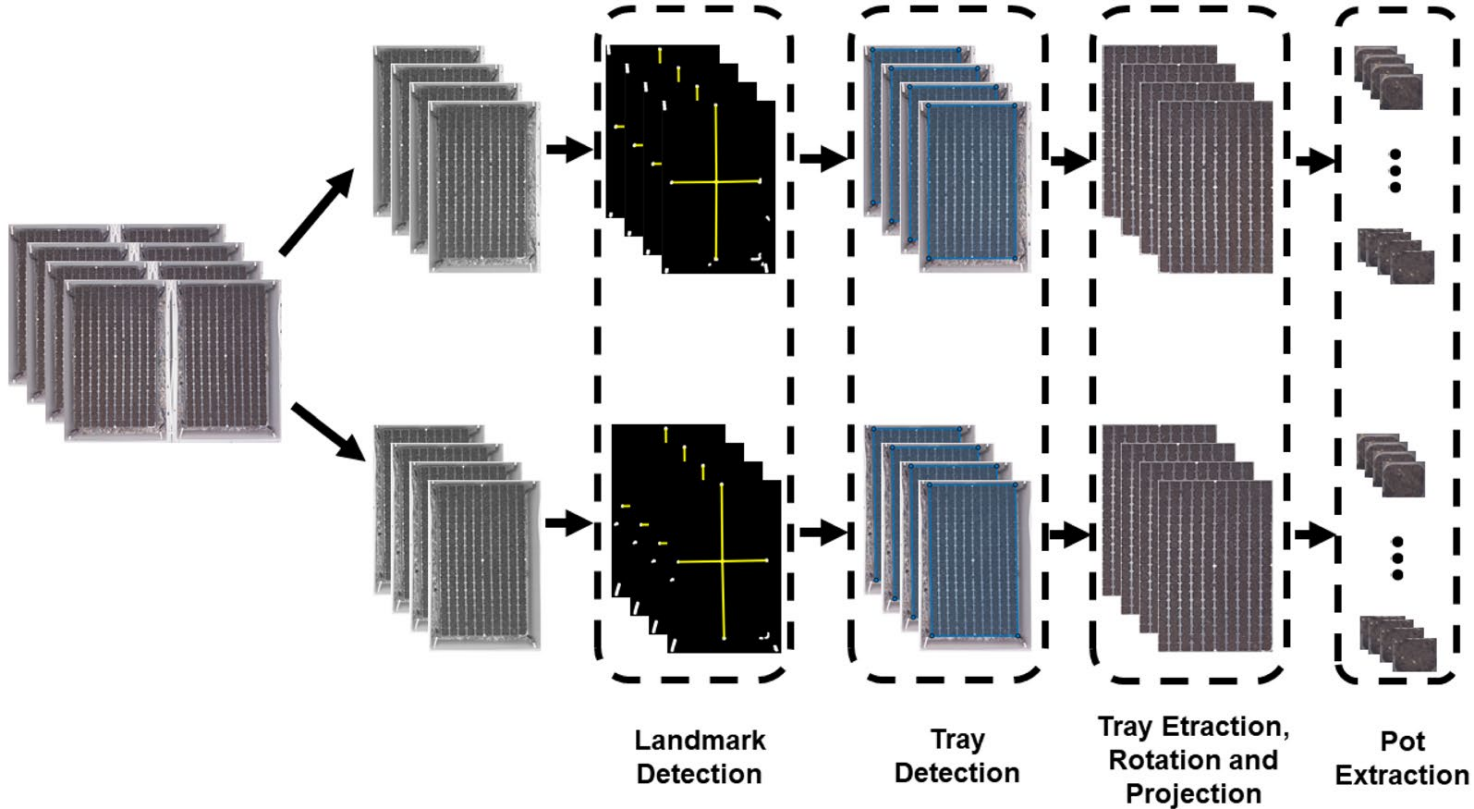
Pot extraction workflow

Since deep learning methods have to predict the seedling developmental stage on an individual basis, the raw images of Fig. 2 could not be directly applied to the neural networks. Thus, the first step of pre-processing was to extract produced crops of each pot. In order to extract them, we needed first to detect, extract, and adjust trays; then, pots were extracted from trays. Figure 4 shows a workflow of the pot extraction from trays, which includes three steps described here below.

#### Landmark detection

In this experiment, trays used included five white landmarks located at the center and four corners of the trays. Because of the constant control of lighting conditions, these five landmarks were detected with a fixed threshold. Then, the five most prominent objects were kept, and the possible remaining small objects were removed. Among the five significant landmarks, the most central object in the images was considered as the central landmark. At the next steps, the four other landmarks were detected based on their minimum angle corresponding to the central landmark with horizontal and vertical axes.

#### Tray detection and extraction

In this step, coordinates of the trays were detected using to the landmarks. Then, based on the coordinates of these landmarks, trays could be extracted from the image. Since trays may not be positioned precisely along the axis of the vertical and horizontal axis sensor of the camera, the trays need to be rotated. The orientation of the trays was found after the computation of the angle of the first eigenvector in the principal component analysis of the modulus of the Fourier transform [29]. Finally, a geometric transformation algorithm [30] was implemented to project the rotated trays to make them straight.

#### Pot extraction

In the last step, all 200 pots of each tray were extracted as an independent temporal sequence of images by using a sliding window with a stride of one pot. The size of these sliding windows was made adjustable by the user to fit with the size of the pot.

This pre-processing pipeline of Fig. 4 has some generic value. Since we did not find something equivalent in the literature for our purpose, we decided to make it available as supplementary material under the form of a free executable (https://uabox.univ-angers.fr/index.php/s/HJAHp0bhZv1zy1j). We believe that despite the simplicity of principle this can be used as a useful tool for any imaging of traits.

### Deep learning methods

The three plant events plus soil (Soil, FA, OC, and FL) to be detected were expected to occur in a definite order. Different supervised strategies to take benefit from this ontological prior-knowledge on the development were tested against the manually established ground-truth as described in the following subsection.

#### Baseline multi-class CNN

As a naive baseline approach, we designed a convolutional neural network (CNN) architecture to predict the classes of each event of Soil, FA, OC, and FL of each frame of the time-lapses independently and without any additional information regarding the temporal order in which they should occur. Given a training set including *K* pairs of images $x_i$ and labels ${\hat{y}}_i$, we trained the parameters $\theta$ of the network *f* using stochastic gradient descent to minimize empirical risk

1 $$\begin{aligned} \theta ^* = \arg \min _{\theta } \sum _{i=1}^K {{\mathcal {L}}} ({\hat{y}}_i, f(x_i, \theta )) \end{aligned}$$

where ${{\mathcal {L}}}$ denotes the loss function, which was chosen as cross-entropy in our case. The minimization was carried out using the ADAM optimizer [31] with a learning rate of 0.001.

**Fig. 5:**
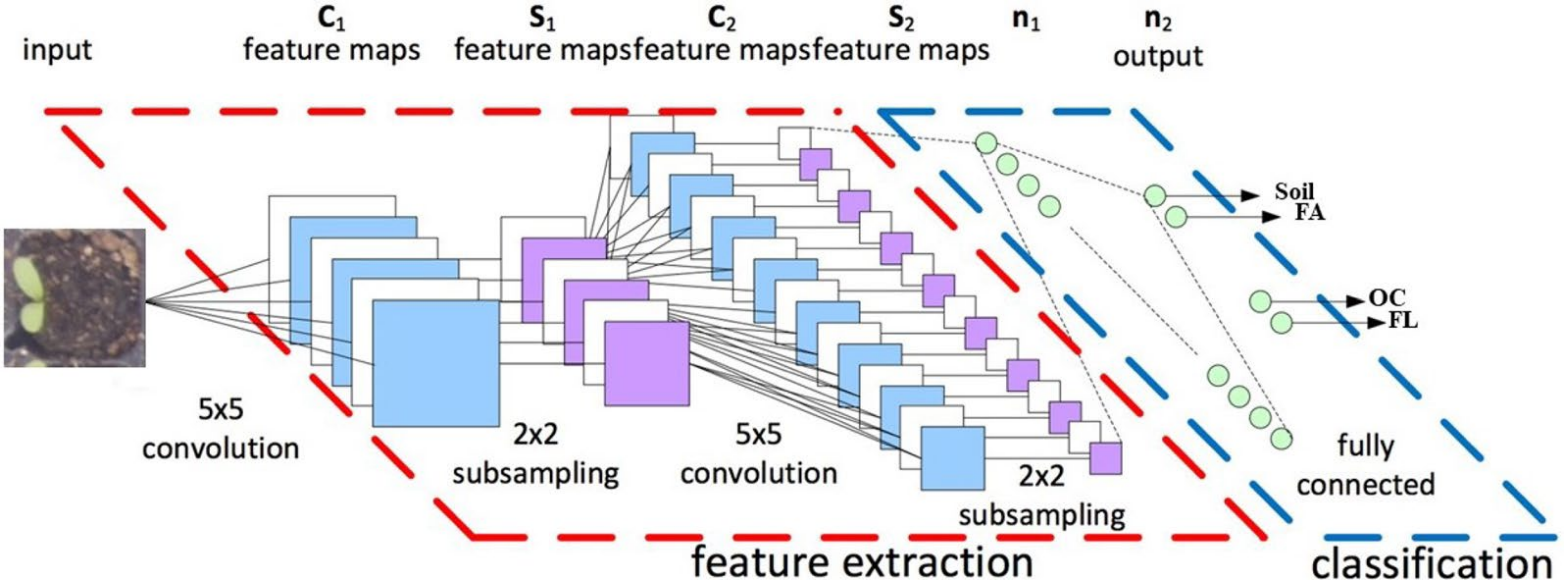
Proposed Muti-class CNN architecture designed to serve as baseline method for the independent classification of each frame of the time-lapses into one of the three stages of plant growth plus soil (Soil, FA, OC, and FL) without any prior temporal order information

Our proposed architecture $f(\cdot ,\cdot )$, shown in Fig. 5, consisted of two main blocks, the feature extraction block, followed by classification block. In a CNN model, the feature extraction block takes care of extracting features from input images by convolutional layers, and the classification block decides classes. Several CNN architectures have been deployed. First, we designed a small AlexNet [32] like CNN structure to keep the number of parameters to be learned low. This AlexNet like CNN is illustrated in Fig. 5 and reads as follows: four convolutional layers with filters of size $3{\times }3$ and respective numbers of filters 64, 128, 256, and 256 each followed by rectified linear unit (RelU) activations and $2{\times }2$ max-pooling; a fully connected layer with 512 units, ReLU activation and dropout (p = 0.5) and a fully connected output layer for four classes corresponding to each event with a softmax activation. We also tested some other well-known larger CNN architectures such as VGG16 [33], Resnet50 [34], and DenseNet121 [35] on our data and choose the one with the highest performance as the base line for a naive memoryless multiclass architecture. These proposed CNN architectures have been optimized on a hold-out set.

#### 2-class CNN’s

The baseline multi-class CNN architecture of Fig. 5 is naive because it does not incorporate the prior knowledge of the ontology of plant growth to decide between different growth steps of plants plus soil (Soil, FA, OC, and FL). As a first improvement of the previous naive baseline, we implemented a variant of the CNN model of Fig. 5 dedicated to the binary classification of two consecutive stages of development. We thus trained 3 models detecting between $M_1$(Soil, FA), $M_2$(FA,OC) and $M_3$(OC,FL). At the beginning of the analysis of an entire time-lapse sequence $M_1$ is used. Then when a first *FA* is detected $M_2$ is applied, and so on until the first FL detection is reached.

#### CNN followed by Long short-term memory

The 2-class CNN’s includes the prior knowledge of the ordered development of the seedling along with a given ontology. However, this prior knowledge is added on top of the CNN. In order to bring a memory directly inside the CNN model, the Long-Short Term Memory (LSTM) architecture was embedded between the feature extraction block and the classification block of the proposed CNN model. LSTM has been proposed [36, 37]. LSTM as a special RNN structure has proven stable and powerful for long-range modeling dependencies in various previous studies [37–39]. The major innovation of LSTM is its memory cell $c^{t}$, which essentially acts as an accumulator of the state information. The cell is accessed, written, and cleared by several self-parameterized controlling gates. Every time a new input comes, its information will be accumulated to the cell if the input gate $i^{t}$ is activated. Also, the prior cell status $c^{t-1}$ could be “forgotten” in this process if the forget gate $f^{t}$ is on. Whether the latest cell output $c^{t}$ will be propagated to the final state $h^{t}$ is further controlled by the output gate $o^{t}$. One advantage of using the memory cell and gates to control information flow is that the gradient will be trapped in the cell [37] and be prevented from vanishing too quickly. In a multivariate LSTM structure, the input, cell output, and states are all 1*D* vectors features from the feature extraction block of the proposed CNN model. The activations of the memory cell and three gates are given as

2 $$\begin{aligned} i^{t} & =  \sigma (W_{xi}x^{t}+W_{hi}h^{t-1}+W_{ci}c^{t-1}+b_{i})\nonumber \\ f^{t} & = \sigma (W_{xf}x^{t}+W_{hf}h^{t-1}+W_{cf}c^{t-1}+b_{f})\nonumber \\ c^{t} & = f^{t}c^{t-1}+i^{t}tanh(W_{xc}x^{t}+W_{hc}h^{t-1}+b_{c})\nonumber \\ o^{t} & = \sigma (W_{xo}x^{t}+W_{ho}h^{t-1}+W_{co}c^{t-1}+b_{o})\nonumber \\ h^{t} & =  o^{t}tanh(c^{t}) \end{aligned}$$

where $\sigma ()$ is the sigmoid function, all the matrices *W* are the connection weights between two units, and $x = (x^{0}, . . . , x^{T-1})$ represents the given input.

**Fig. 6:**
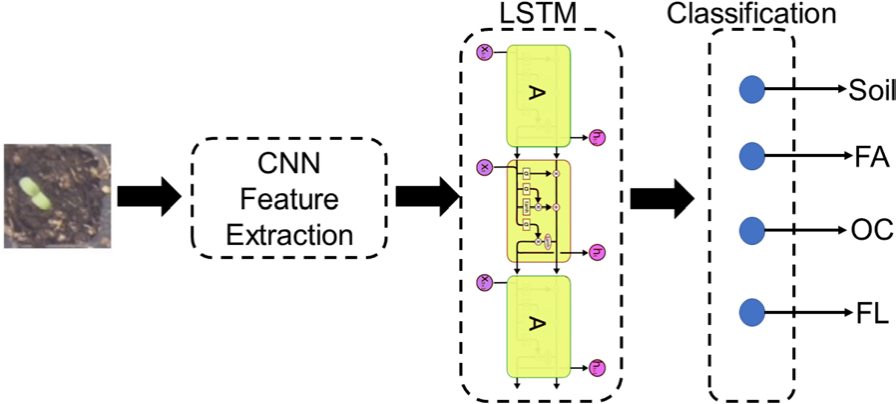
CNN-LSTM block

The CNN-LSTM architecture is an integration of a CNN (Convolutional layers) with an LSTM. First, the CNN part of the model process the data and extract features then the one-dimensional feature vectors feed to an LSTM model to support sequence prediction. CNN-LSTMs are a class of models that is both spatially and temporally deep and has the flexibility to be applied to a variety of vision tasks involving sequential inputs and outputs. Fig. 6 shows a schematic of a CNN-LSTM model.

The proposed CNN-LSTM model consisted of the same convolutional layers as the multi-class CNN model of Fig.4 and an LSTM layer with 128 units.

#### Convolutional LSTM (ConvLSTM)

As an alternative to CNN-LSTM, we use ConvLSTM [40] which has convolutional structures in both the input-to-state and state-to-state transitions. In ConvLSTM all the inputs $X^{1}; \cdot \cdot \cdot ;X^{t}$, cell outputs $C^{1}; \cdot \cdot \cdot ; C^{t}$, hidden states $H^{1}; \cdot \cdot \cdot ; H^{t}$, and gates $i^{t}$; $f^{t}$; $o^{t}$ of the ConvLSTM are 3D tensors whose last two dimensions are spatial dimensions (rows and columns). The ConvLSTM determines the future state of a certain cell in the grid by the inputs and past states of its local neighbors. This can easily be achieved by using a convolution operator in the state-to-state and input-to-state transitions. The key equations of ConvLSTM are shown in 3 below, where ‘$\circledast$’ denotes the convolution operator.

3 $$\begin{aligned} i^{t} & =  \sigma (W_{xi} \circledast x^{t}+W_{hi} \circledast h^{t-1}+W_{ci}c^{t-1}+b_{i})\nonumber \\ f^{t} &= \sigma (W_{xf} \circledast x^{t}+W_{hf} \circledast h^{t-1}+W_{cf}c^{t-1}+b_{f})\nonumber \\ c^{t} & = f^{t}c^{t-1}+i^{t}tanh(W_{xc}\circledast x^{t}+W_{hc} \circledast h^{t-1}+b_{c})\nonumber \\ o^{t} & = \sigma (W_{xo}\circledast x^{t}+W_{ho}\circledast h^{t-1}+W_{co}c^{t-1}+b_{o})\nonumber \\ h^{t} & = o^{t}tanh(c^{t}) \end{aligned}$$

**Fig. 7:**
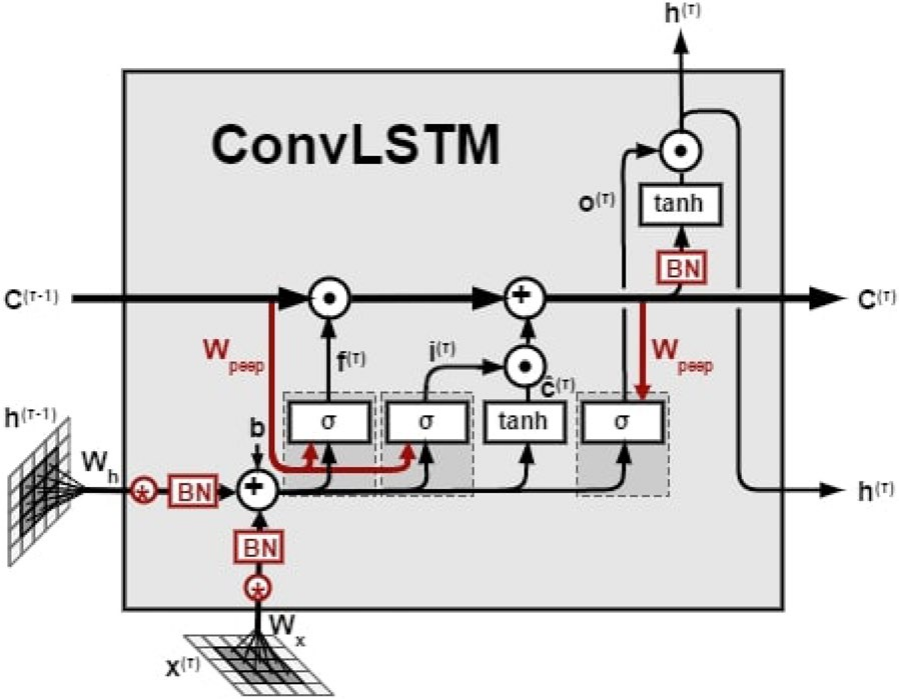
ConvLSTM block with one cell [40]

Figure 7 shows a schematic of the ConvLSTM method adopted for our purposes.

#### Post-processing

The passing from one developmental stage to another can consist of very tiny details. This was, for instance, the case for FA and FL in our case. To address this problem, a post-processing smoothing filter can be designed to reduce the fluctuations that may appear when the seedling shift from one developmental stage to another. Also, post-processing can be of help when the first leaf moves out of the frame after a period of time and just cotyledons remain in the frame in each individual pot. In this case, the model just sees cotyledons and without post-processing would predict a label corresponding to the OC stage. Post-processing can be designed to prevent some switches forbidden by the developmental ontology and in this case keep the stage of the growth at FL.

**Fig. 8:**
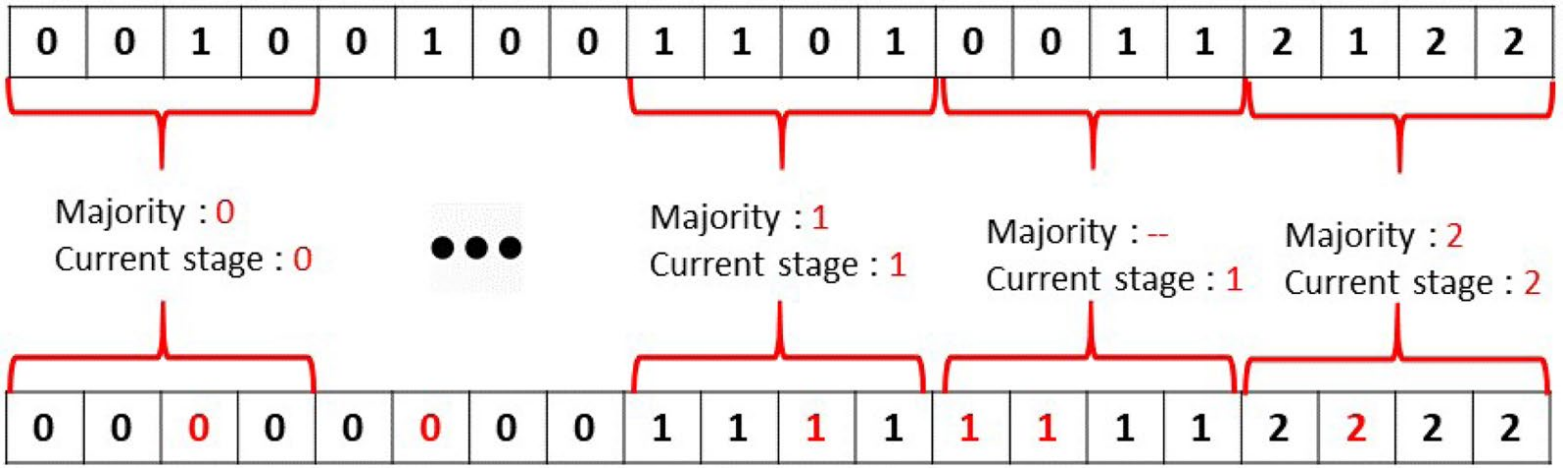
An example of the post-processing step on predicted classes where the sliding window size is four images

The designed post-processing smoothing filter illustrated in Fig. 8 was based on a sliding window computing a majority voting by finding the median of classes (4)

4 $$\begin{aligned} c = {\lfloor }*{\rfloor }{\left\{ \left( \frac{n+1}{2} \right) \right\} ^{th}} \end{aligned}$$

where *c* and *n* represent predicted class and window size, respectively. Additionally, this window replaced the current stage of all neighbors to all labels that detected as the previous stage.

**Fig. 9:**
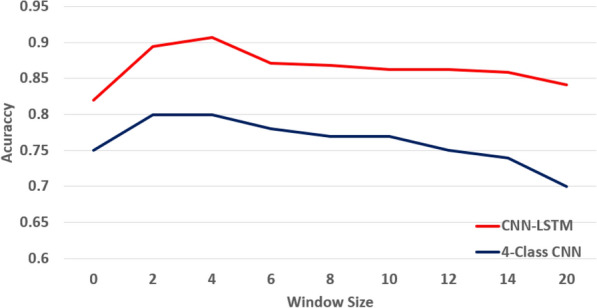
Classification accuracy as a function of denoising windows size

The size of the sliding window was optimized on the CNN-LSTM and multi-class CNN architecture. As shown in Fig. 9, performances were found optimal for both architectures on the training data set for a size of 4 frames, corresponding to an observation of 1 hour in our case.

## Results and discussion

First, we compared the performance of the tested CNN multi-class structures as shown in Table 2. As expected the performance of deeper architectures like ResnNet50 and DenseNet121 is less than smaller deep models such as our proposed model or VGG16. Indeed, increasing parameters in a CNN model lead to over-fitting due to low image dimensions and limited variability in the database [41]. For the following, we keep the best multi-class structure (our proposed CNN of Fig. 5) as baseline model to be compared with other architectures including temporal information.

**Table 2:** The average performance of baseline multi-class CNN models with different evaluation metrics on images without soil background

Model	Accuracy	Error	Sensitivity	Specificity	Precision	False positive rate
Proposed CNN	0.80 ± 0.19	0.20 ± 0.19	0.85 ± 0.13	0.93 ± 0.07	0.85 ± 0.14	0.07 ± 0.07
VGG16	0.80 ± 0.24	0.2 ± 0.24	0.84 ± 0.18	0.93 ± 0.12	0.85 ± 0.07	0.07 ± 0.11
ResNet50	0.78 ± 0.18	0.22 ± 0.18	0.77 ± 0.21	0.89 ± 0.09	0.85 ± 0.11	0.08 ± 0.05
DenseNet121	0.79 ± 0.09	0.21 ± 0.09	0.78 ± 0.08	0.90 ± 0.14	0.86 ± 0.09	0.07 ± 0.10

The proposed deep learning methods multi-class CNN, 2-class CNN’s, CNN-LSTM, and ConvLSTM were applied to the dataset produced by our imaging system after pre-processing and post-processing as described in the previous section. We now present and discuss the associated results. The performances of the different deep learning methods tested on our dataset were assessed with classical metrics such as accuracy, error, sensitivity, specificity, precision, and false alarm positive rate. They are provided in Tables 3 and 4, respectively, for images with and without soil background.

**Table 3:** The average performance of models with different evaluation metrics on images with soil background

Model	Accuracy	Error	Sensitivity	Specificity	Precision	False positive rate
Multi-class CNN	0.63 ± 0.20	0.37 ± 0.20	0.63 ± 0.2	0.94 ± 0.05	0.88 ± 0.1	0.06 ± 0.05
2-class CNN’s	0.72 ± 0.25	0.28 ± 0.26	0.72 ± 0.24	0.95 ± 0.06	0.90 ± 0.11	0.08 ± 0.05
CNN-LSTM	0.83 ± 0.10	0.15 ± 0.10	0.82 ± 0.10	0.93 ± 0.06	0.85 ± 0.10	0.06 ± 0.06
ConvLSTM	0.62 ± 0.2	0.33 ± 0.2	0.68 ± 0.2	0.93 ± 0.07	0.84 ± 0.1	0.06 ± 0.06

Tables 3 and 4 show that all methods performed better than the naive multi-class CNN architecture, which was processing the temporal frames independently of any prior knowledge on the order of the ontological development of seedling. The best strategy to incorporate this knowledge among the ones tested was found to be the CNN-LSTM architecture, which outperforms all other models for all tested metrics. Removing the soil numerically, clearly improves all methods while keeping the CNN-LSTM architecture as the best approach.

**Table 4:** Average performance of models on images without soil background

Model	Accuracy	Error	Sensitivity	Specificity	Precision	False positive rate
Multi-class CNN	0.80 ± 0.19	0.20 ± 0.19	0.85 ± 0.13	0.93 ± 0.07	0.85 ± 0.14	0.07 ± 0.07
2-class CNN’s	0.88 ± 0.18	0.12 ± 0.18	0.86 ± 0.10	0.95 ± 0.05	0.86 ± 0.11	0.05 ± 0.05
CNN-LSTM	0.90 ± 0.08	0.10 ± 0.07	0.87 ± 0.11	0.96 ± 0.03	0.88 ± 0.15	0.04 ± 0.04
ConvLSTM	0.81 ± 0.11	0.21 ± 0.09	0.85 ± 0.03	0.92 ± 0.09	0.85 ± 0.12	0.07 ± 0.10

Our experimental results show that a reasonable recognition rate of plant growth stages detection (approximately $90\%$) can be achievable by the CNN-LSTM model. Additionally, we measured the performance of our best model (CNN-LSTM) and on worst model (multi-class CNN) on test data before and after post-processing. Table 5 shows that the metrics of performance are systematically improved by a significant 5 to 8%.

**Table 5:** Average performance of the baseline multi-class CNN and best trained models (CNN-LSTM) on test data before and after post-processing step

Model	Accuracy	Error	Sensitivity	Specificity	Precision	False positive rate
Multi-class CNN (Before)	0.72 ± 0.29	0.28 ± 0.29	0.73 ± 0.19	0.94 ± 0.21	0.91 ± 0.13	0.8 ± 0.08
Multi-class CNN (After)	00.80 ± 0.19	0.20 ± 0.19	0.85 ± 0.13	0.93 ± 0.07	0.85 ± 0.14	0.07 ± 0.07
CNN-LSTM (Before)	0.84 ± 0.04	0.16 ± 0.04	0.83 ± 0.05	0.93 ± 0.06	0.86 ± 0.09	0.05 ± 0.05
CNN-LSTM (After)	0.90 ± 0.08	0.10 ± 0.07	0.87 ± 0.11	0.96 ± 0.03	0.88 ± 0.15	0.04 ± 0.04

It is possible to have a more in-depth analysis of the remaining errors by looking at the confusion matrix of this CNN-LSTM model, as given in Table 6. This confusion matrix shows that most of the errors, almost 98%, happen between the most complicated classes of OC and FL while the remaining 2% of errors appear on borders of the first two classes of soil and FA. The confusion matrix helps us to analyse the performance of the trained model on each individual class. The F1-score of Eq. (5) is considered as one of the common metrics to analyse confusion matrices for each class by calculating the harmonic mean of precision and recall (Table 6 right) where TP, FP, and FN stands for True Positive, False Positive, and False Negative respectively. It shows that the trained model can perform better on the first two classes of Soil and FA with the highest scores of 0.98 and 0.90 on predicted data while the class of OC is the most challenging class.

5 $$\begin{aligned} F1-score = \times \frac{Precision \times Recall}{Precision + Recall} = \frac{2\times TP}{2\times TP + FP + FN} \end{aligned}$$

**Table 6:** Confusion matrix and F1-score of cross-subject performance where the best deep learning method, the CNN-LSTM architecture is used

True classes	Predicted					
	Soil	FA	OC	FL		F1-Score
Soil	97531	0	0	0	Soil	0.98
FA	2591	*26855*	2915	0	FA	0.90
OC	0	0	*58668*	19556	OC	0.79
FL	0	0	8219	*90610*	FL	0.87

In order to evaluate the robustness and transferability of the best trained model (CNN-LSTM), an additional test was done on images of 50 pots of another genotypes of the red clove species which were captured from a new experiment. Table 7 shows that the average classification accuracy on the new genotype are very close to the one obtained with alfalfa. This confirms the transferability and robustness of the model from one genotype to another.

**Table 7:** Average performance of the trained models on images of new genotype of red cloves as well as the species of alfaalfa

Model	Accuracy	Error	Sensitivity	Specificity	Precision	False positive rate
CNN-LSTM(red cloves)	0.91 ± 0.01	0.09 ± 0.01	0.88 ± 0.05	0.96 ± 0.02	0.86 ± 0.08	0.04 ± 0.03
CNN-LSTM (alfalfa)	0.90 ± 0.08	0.10 ± 0.07	0.87 ± 0.11	0.96 ± 0.03	0.88 ± 0.15	0.04 ± 0.04

**Fig. 10:**
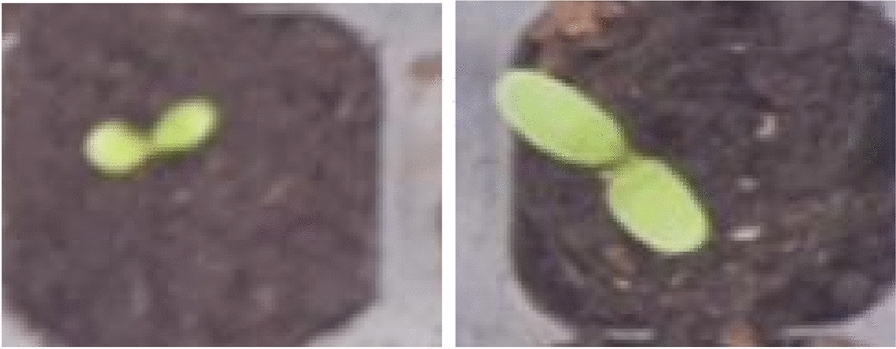
A sample of images from two plant species used for training (left) and testing (right) dataset

One may wonder where the classification errors in this experiment can come from. In our error analyses, we found four different sources of errors in the experiment. The first source of errors can come from the different cotyledons and leaf sizes of the two species, as the cotyledons and leaf size of a species can be much bigger or smaller compared with other species. Usually, this type of error happens in the borders of two classes of OC and FL. Figure 10 shows an example of these differences in the size of two plant species. Data augmentation with a variation on the zoom could be a solution to help with these errors.

The second source of errors can be due to the circadian cycle of plants during the growth. The circadian cycle of plants makes some movements on cotyledon and leaves during day and nights [42]. This type of error can happen at the border of FA and OC, where these movements make a delay for the detection of fully opening cotyledon. Also, this type of error can happen at the border of two classes of OC and FL, where the circadian cycle does not allow the system to recognize the appearance of the first leaf from the middle of the cotyledon. The third source of errors happens due to the overlapping of plants in a tray. Plants grow at different speeds and directions in a tray, and it makes overlapping on plants of neighbor pots at some points. This type of error usually happens in the last two classes of OC and FL. The last source of the errors can come from annotation errors. In general, the annotation of plant growth stages is challenging since plants grow continuously; it means there are no striking events of growth. In this case, a class represents a period of growth. For instance, the FA class is assigned to images which were captured in the period of the first appearance of the cotyledon till the time of the fully opening of the cotyledon. In this case of annotation, different annotators may define the ending of a stage period with an approximate delay of 15 images. Also, there is a period of formation of the first leaf before its unfolding during plant growth. This period is considered to be a part of the FL class in this experiment. This consideration may bring an additional error for annotation of stages as different annotators may recognize the beginning of the leaf formation with a delay.

## Conclusion and perspectives

In this paper, we have presented a complete image processing and machine learning pipeline to classify three stages of plantlet growth plus soil on the different accessions of two species of red clover and alfalfa.

Different strategies were compared in order to incorporate the prior information of the order in which the different stages of the development occur. The best classification performance on these types of images was found with our proposed CNN-LSTM model, which achieved 90% accuracy of detection with the help of a denoising algorithm incorporating the ontological order in the development stages.

In our experiments all models were trained and tested on several genotypes of two species of red clover and alfaalfa. Presented results shows that trained model is robust on some genotypes but it does not guaranty the robustness of the model an all genotypes or other species. In order to increase the robustness of models one could either add more real data from several genotypes or use data augmentation to synthetically increase the data variability in the training database [43–45] based on possible priors on the expected morphological plasticity of the species.

These results can now be extended in various directions. It will be interesting to extend the approach to a range of species of agricultural interest in order to provide a library of trained networks. From this perspective, it could be interesting to investigate quantitatively how, by their similarity in shape, the knowledge learned on some species could be transferred to others via transfer learning, domain adaptation, or hierarchical multi-label classification [46]. More events of the development of plants could also be added to extend the investigation of seedling kinetics. This includes for instance the instant where cotyledons are out of soil fully or rise of the first leaf before unfolding. These extensions could be tested easily following the global methodology presented in this article to assess the deep learning models. For even more advanced stages of development and yet still accessible from top view, the issue of plant overlapping each other would arise and become a limitation. Solving this would require to switch to tracking algorithms in order follow and label the trajectory of each plant despite ambiguity created by partial occlusion and overlapping. Other deep learning architectures would have to be tested in this perspective [47]. As another possible direction, in this study, since we used classical standard RGB images, plants were not measured during nights, and some missed events could shift the estimation of the developmental stages of the seedlings. Lidar cameras, accessible at low-cost [48], could be used to access to night events. Also, Bayesian approaches [6], such as Gaussian processes, could be used to estimate the time for the possibly missing information.

## Data Availability

Data will be available after acceptance upon reasonable request
